# A Rescue Strategy for Handling Unevaluable Patients in Simon’s Two Stage Design

**DOI:** 10.1371/journal.pone.0137586

**Published:** 2015-09-14

**Authors:** Lisa Belin, Philippe Broët, Yann De Rycke

**Affiliations:** 1 Biostatistics department, Hôpital, Institut Curie, Paris, France; 2 UMR 1178, INSERM, Villejuif, France; 3 Université Paris Sud, Villejuif, France; 4 Hôpital Paul Brousse, AP-HP, Villejuif, France; 5 Epidemiology and Clinical Research department, Hôpital Bichat, AP-HP, Paris, France; 6 Centre de pharmacoépidémiologie (Cephepi), Hôpital Bichat, AP-HP, Paris, France; 7 UMR 1123 ECEVE, Université Paris Diderot, Sorbonne Paris Cité, Paris, France; 8 UMR 1123 ECEVE, INSERM, Paris, France; 9 CIC-EC 1425, INSERM, Paris, France; Yale School of Public Health, UNITED STATES

## Abstract

For phase II oncology trials, Simon’s two-stage design is the most commonly used strategy. However, when clinically unevaluable patients occur, the total number of patients included at each stage differs from what was initially planned. Such situations raise concerns about the operating characteristics of the trial design. This paper evaluates three classical *ad hoc* strategies and a novel one proposed in this work for handling unevaluable patients. This latter is called the *rescue* strategy which adapts the critical stopping rules to the number of unevaluable patients at each stage without modifying the planned sample size. blue Simulations show that none of these strategies perfectly match the original target constraints for type I and II error rates. Our *rescue* strategy is nevertheless the one which best approaches the target error rates requirement. A re-analysis of one real phase II clinical trials on metastatic cancer illustrates the use of the proposed strategy.

## Introduction

In oncology, phase II studies are used to screen novel therapies with anti-tumoral activity and to determine if they offer sufficient clinical interest to justify initiating a large-scale phase III trial. Decision rules are usually based on short-term binary endpoints such as tumor response evaluated at a given clinically relevant time-point: patients are classified as either responders or non-responders. The first phase II design was proposed by Gehan [1] followed by other multistage designs proposals (see for example: Simon [2], Fleming [3]). The Simon’s two-stage design [2] is nowadays the most widely used strategy [4–6]. Lee *et al*. [7] reported that one-fifth of randomized phase II trial, with a reported statistical design, is a Simon two-stage design. This design allows early stopping due to lack of treatment efficacy during the first stage. Otherwise, additional patients are included for the second stage, at the end of which a conclusion can be drawn as to the futility or efficacy of the treatment. Estimation of the magnitude of the treatment effect is a secondary objective. Simon’s two-stage designs allow to determine the sample size and the critical stopping values at each stage. The critical stopping values are established for rejecting a null hypothesis with control of pre-specified type I and type II error rates.

In practice, unevaluable patients at the time of analysis are nevertheless expected to occur. Here, an unevaluable patient is defined as a patient whose response to treatment cannot be determined due to the occurrence of some concurrent uncontrolled event which modifies the therapeutic evaluation schedule. Non-evaluability may occur despite the efforts of the investigators to follow the evaluation schedule as carefully as possible. Such situation is exemplified in our clinical example where the Magnetic resonance imaging (MRI) exam can be postponed for several weeks due to the critical condition of the patient. In some cases, the non evaluability can be informative such as the occurence of a toxicity that requires to stop the treatment. In this work, we will only consider non informative unevaluable patients occurrence.

Due to the occurrence of unevaluable patients, the actual sample size of the first or second stage may differ from what has been originally planned. In routine practice, various *ad hoc* strategies are used for handling these unevaluable patients such as: (i) to consider unevaluable patients as non-responders, (ii) to exclude the unevaluable patients and re-estimate the boundaries for the actual sample size, and (iii) to include new patients in order to achieve the planned sample size. To the best of our knowledge, these three *ad hoc* strategies are used without a detailed knowledge of their consequences on the original targets for type I and II error rates.

The main objective of this study is to evaluate how the overall type I and type II error rates are affected by unevaluable patients. We investigate the three classical strategies previously described and a novel one proposed in this work. This latter strategy resets the critical stopping rules for taking into account patient availability at the study endpoint. We call it a *rescue* strategy since it aims at best minimizing deviations from planned error rates without changing the initial sample size.

## Presentation of the strategies

### Simon design and notation

The main principles and objectives of a Simon’s phase II design are summarized hereunder.

Let *t*_0_ be the clinically relevant time-point for therapeutic evaluation specified by the investigators in the protocol.

Let *π* denote the true response rate for the treatment at *t*_0_. The null hypothesis is: *H*_0_ : *π* ≤ *π*_0_ where *π*_0_ represents an uninteresting level of treatment efficacy at *t*_0_. The alternative hypothesis is: *H*_1_ : *π* ≥ *π*_1_ where *π*_1_ represents the desirable target level of treatment efficacy evaluated at *t*_0_. The aim of Simon’s two-stage design is to satisfy the pre-specified type I (*α*) and type II (*β*) error rates together.. For testing *H*_0_ : *π* ≤ *π*_0_ *vs* *H*_1_ : *π* ≥ *π*_1_, a Simon’s two-stage design is usually indexed by four numbers (*r*_1_, *n*_1_, *r*_2_, *n*_2_). It indicates that at the first stage *n*_1_ patients are included, the total sample size is of *n*_2_ and (*r*_1_, *r*_2_) are the stopping boundaries at stage 1 and 2 respectively. If *r*_1_ or fewer responses among *n*_1_ patients are observed, the trial is terminated and the null hypothesis is not rejected. If more than *r*_1_ responses are observed *n*_2_ − *n*_1_ additional patients are included. If the total number of observed responses among the total *n*_2_ patients is less or equal to the stopping boundary *r*_2_, the null hypothesis is not rejected and the trial is not recommended for further studies. If more than *r*_2_ responses are observed, we reject the null hypothesis and conclude that the treatment has shown efficacy.

In the following, we denote *X*_*j*_ the number of responses among *n*_*j*_ patients at the stage *j* (*j* = 1, 2), $X_{j}=\overset{n_{j}}{\underset{i=1}{⅀}}R_{i}$ with *R*_*i*_ being the indicator function of a response for patient *i* (*i* = 1, …, *n*_*j*_). Under the null hypothesis, *R*_*i*_ follows a Bernoulli distribution with probability *π*_0_.

In his seminal work [2], Simon introduced the probability of rejecting the null hypothesis when the treatment efficacy is *π* as:

$$A(\pi )=P(\{X_{1}>r_{1}\}\cap \{X_{2}>r_{2}\}|\pi ,n_{1},n_{2}).$$

To establish Simon’s two-stage design, two strategies are usually considered: these are the optimal and the minimax design strategies. Among all the designs satisfying the constraints *A*(*π*_0_) ≤ *α* and *A*(*π*_1_) ≥ 1 − *β*, the minimax design minimizes the maximal sample size (*n*_*j*_) and then given the value of *n*_*j*_ minimize the average sample size under the null hypothesis. The optimal design minimizes the expected sample size under the null hypothesis such as:

$$E(n_{2}|H_{0})=n_{1}+[1-P(X_{1}\leq r_{1}|H_{0})](n_{2}-n_{1}).$$

For specified values of *n*_1_, *n*_2_, *π*_0_, *π*_1_, *α* and *β*, one determine the stopping boundaries *r*_1_ and *r*_2_. In case where no stopping boundaries could be found, the investigator can increase either the total sample size or increase type I and type II error rate.

In the following, we present the three *ad hoc* strategies for situations with *Z*_*j*_ unevaluable patients at stage *j*.

### Ad hoc strategies

The three classical *ad hoc* strategies to manage unevaluable patients are:

- The *maximum bias* strategy which considers an unevaluable patient *i* as being a non-responder such that *R*_*i*_ = 0.
- The *exclusion* strategy which excludes unevaluable patients from the set of enrolled patients but modifies the stopping boundaries to reflect the loss of information due to unevaluable patients. Then, the number of observed responses over the evaluable patients (*n*_*j*_ − *Z*_*j*_) is compared to *$\frac{n_{j}-Z_{j}}{n_{j}}r_{j}$* rounded to the nearest integer. These new stopping boundaries take into account the observed fraction of evaluable patients.
- The *replacement* strategy which includes additional patients to reach the planned sample size.

However, the first one is expected to give biased estimate of the response rate and could strongly penalized the treatment. The *exclusion* strategy may be unbiased but loss of information could lead to deviations in terms of type I and type II error rates. Finally, the *replacement* strategy requires a particular monitoring to include new patients as soon as one patient is classified as unevaluable. Facing these issues, a new strategy is proposed.

### The proposed strategy

The proposed strategy is a *rescue* strategy so that the planned sample size is unmodified and no additional patients than planned is included. The proposed *rescue* strategy is based on computing new stopping boundaries that take into account the number of evaluable patients observed at each stage. If no stopping boundaries satisfy the initial requirements, an increase of type I (*α*) and type II (*β*) error rates is considered. The computation of these new stopping boundaries relies on the conditional probability of responding at *t*_0_ for an evaluable patient at each stage under the null and the alternative hypothesis. We give below the rationale and the methods for computing these boundaries.

#### General Framework

For each patient *i*, let *T*_*i*_ be a latent failure time which underlies the observed binary outcome *R*_*i*_ such as: *R*_*i*_ = 1 if *T*_*i*_ > *t*_0_ (therapeutic success) and *R*_*i*_ = 0 otherwise (therapeutic failure). Let *C*_*i*_ be a latent censoring time and assume that *T*_*i*_ and *C*_*i*_ satisfy the condition of independent censoring [8]. Moreover, the outcome *R*_*i*_ is unknown for situations where {*C*_*i*_ < *T*_*i*_} ∩ {*C*_*i*_ < *t*_0_} which corresponds to a situation with an unevaluable patient. We denote *F*_*kj*_(*t*), *f*_*kj*_(*t*), *G*_*kj*_(*t*), *g*_*kj*_(*t*) the cumulative distribution function and the probability density function for *T* and *C*, respectively.

The new stopping boundaries for the number of evaluable patients (*n*_*j*_ − *Z*_*j*_) are computed in the same way as the classical Simon procedure. However the Bernoulli parameter of variables *R*_*i*_ is no longer *π*_*k*_ but $\pi_{kj}^{*}$ that corresponds to the conditional probabilities of responding at *t*_0_ for an evaluable patient at each stage (*j* = 1, 2) under the null and the alternative hypothesis (*k* = 0, 1). The key parameters of the *rescue* strategy are $\pi_{kj}^{*}$ (see mathematical demonstration in S1 Text for more details):

$$\begin{array}{ccc} \pi_{kj}^{*} & = & P\;(\{T>t_{0}\}|\bar{\left\{C<T\}\cap \{C<t_{0}\right\}})\\ & = & 1-\frac{\tau_{kj}^{*}}{1-P(\{C<T\}\cap \{C<t_{0}\})}, \end{array}$$ (1)

with $\tau_{kj}^{*}$ the probability of not responding to the therapy and being evaluable at *t*_0_

$$\tau_{kj}^{*}=P(\{T<t_{0}\}\cap \{T<C\}),$$

and *P*({*C* < *T*} ∩ {*C* < *t*_0_}), the probability of being unevaluable at *t*_0_.

From the cumulative distribution and probability density functions introduced just above, $\tau_{kj}^{*}$ can be expressed such as:

$$\tau_{kj}^{*}=\int_{0}^{t_{0}}F_{kj}(c)g_{kj}(c)dc+F_{kj}(t_{0})[1-G_{kj}\left(t_{0}\right)].$$ (2)

In the following, we assume that *F*_*kj*_(*t*) is a Weibull distribution with shape (*h*_0_*kj*__) and scale (*γ*_*j*_) parameters and *G*_*kj*_(*t*) is a uniform distribution over the interval [**0**; *λ*_*kj*_]where *λ*_*kj*_ is the minimum value which corresponds to the proportion of unevaluable patients. Estimation procedure of the three parameters (*h*_0_*kj*__, *γ*_*j*_, *λ*_*kj*_) will be detailled in the next paragraph.

An estimate of $\tau_{kj}^{*}$ is obtained by substituting the estimated parameters values of these distributions into formula 2.

In practice, we are able to obtain some information for the patient status at intermediate evaluation. If we denote ${\tilde{t}}_{0}$ with (${\tilde{t}}_{0}<t_{0}$) this timepoint, we can obtained estimate of the shape and the scale parameters of the Weibull distribution (*h*_0_*kj*__, *γ*_*j*_)by considering the actuarial survival estimates at stage *j* at two different time-points (${\tilde{t}}_{0}$ and *t*_0_) denoted by: $\left[1-{\hat{F}}_{j}\left({\tilde{t}}_{0}\right)\right]$ and $\left[1-{\hat{F}}_{j}\left(t_{0}\right)\right]$.

Thus, we obtain:

$${\hat{h}}_{0_{kj}}=t_{0}{\left[-ln\left(\pi_{k}\right)\right]}^{-\frac{1}{{\hat{\gamma }}_{j}}},$$

and

$${\hat{\gamma }}_{j}=\frac{ln\left\{\frac{ln\left[1-\hat{F_{j}}\left(t_{0}\right)\right]}{ln\left[1-\hat{F_{j}}\left({\tilde{t}}_{0}\right)\right]}\right\}}{-ln\left(\frac{{\tilde{t}}_{0}}{t_{0}}\right)}.$$ (3)

It is worth noting that if only one time evaluation is available, *γ*_*j*_ is set to unity so that Weibull distribution reduces to an exponential distribution with parameter $[-ln(\pi_{k})]t_{0}^{-1}.$

Then, we obtain an estimate of *λ*_*kj*_ (denoted ${\hat{\lambda }}_{kj}$) by solving:

$$\int_{0}^{+\infty }\frac{min(t,t_{0},\lambda_{kj})}{\lambda_{kj}}{\hat{f}}_{kj}(t)dt=\frac{Z_{j}}{n_{j}},$$ (4)

where the probability of being unevaluable at *t*_0_ is estimated by $\frac{Z_{j}}{n_{j}}$.

Finally, we estimate $\pi_{kj}^{*}$ as:

$${\hat{\pi }}_{kj}^{*}=1-\frac{{\hat{\tau }}_{kj}^{*}}{1-(\frac{Z_{j}}{n_{j}})}$$ (5)

with

$${\hat{\tau }}_{kj}^{*}=\int_{0}^{t_{0}}{\hat{F}}_{kj}(c){\hat{g}}_{kj}(c)dc+{\hat{F}}_{kj}(t_{0})[1-{\hat{G}}_{kj}\left(t_{0}\right)]$$

where ${\hat{F}}_{kj}$ and ${\hat{G}}_{kj}$ are the Weibull and uniform distributions with estimated parameters obtained as presented above.

#### Practical implementation of the *rescue* strategy

We compute the new stopping boundaries $r_{1}^{*1}$ and $r_{2}^{*1}$ that match as close as possible to the initial type I and II error requirements as described in Section Simon design and notation. For this purpose, we propose to explicitly consider (*α*) and (*β*) changes through a specified error rate function. We use an error rate function (denoted thereafter *ϕ*) that allows *α* to increase while *β* is set to keep the ratio $\frac{\beta }{\alpha }$ as close as possible to the initial type I and type II error rates.

To generate stopping boundaries $\left(r_{1}^{*1},r_{2}^{*j}\right)$, this algorithm must be followed:

At each stage *j*,

1. calculate the number of evaluables patients given by (*n*_1_ − *Z*_1_; *n*_2_ − *Z*_*j*_)
2. estimate ${\hat{\pi }}_{0j}^{*}$ and ${\hat{\pi }}_{1j}^{*}$,
3. for each $\left(r_{1}^{*1},r_{2}^{*j}\right)$ with $r_{1}^{*1}\leq n_{1}-Z_{1}$ and $r_{2}^{*j}\leq n_{2}-Z_{j}$, calculate
  $$A({\hat{\pi }}_{0j}^{*})=P(\{X_{1}>r_{1}^{*1}\}\cap \{X_{2}>r_{2}^{*j}\}|{\hat{\pi }}_{0j}^{*},n_{1}-Z_{1},n_{2}-Z_{j})$$
  and
  $$A({\hat{\pi }}_{1j}^{*})=P(\{X_{1}>r_{1}^{*1}\}\cap \{X_{2}>r_{2}^{*j}\}|{\hat{\pi }}_{1j}^{*},n_{1}-Z_{1},n_{2}-Z_{j}),$$
4. select $\left(r_{1}^{*1},r_{2}^{*j}\right)$ as $A({\hat{\pi }}_{0j}^{*})\leq \alpha$ and $A({\hat{\pi }}_{1j}^{*})\geq 1-\beta$.

If several designs $\left(r_{1}^{*1},n_{1}-Z_{1},r_{2}^{*j},n_{2}-Z_{j}\right)$ can be obtained, the selected design is the one which minimize *E*(*n*_2_ − *Z*_*j*_ ∣ *H*_0_).

If no design is obtained, type I and type II error rates are increased and controlled using the error rate function *ϕ*, step 1-2-3 is repeated with these new type I and type II error rates. The final design is the one which minimize the increase of type I and type II error rate.

R functions to implement the *rescue* strategy are available in S2 File.

At the first stage, if the number of responses for *n*_1_ − *Z*_1_ evaluable patients is inferior or equal to $r_{1}^{*1}$, the null hypothesis is not rejected and the trial is stopped for futility. Otherwise, *n*_2_ − *n*_1_ additional patients are included. If no more unevaluable patients are observed the stopping boundary $r_{2}^{*1}$ is used to make the final outcome of the trial. If some additional unevaluable patients are observed at the second stage (*Z*_2_ > *Z*_1_), a new stopping boundary $r_{2}^{*2}$ is computed. If the number of responses for *n*_2_ − *Z*_2_ evaluable patients is inferior or equal to $r_{2}^{*2}$, we conclude to not reject the null hypothesis. If there are more than $r_{2}^{*2}$ the null hypothesis is rejected and trial concludes to treatment efficacy.

## Simulation

### Simulation protocol

#### Latent failure and censoring times

A simulation study was performed in order to mimic realistic situations with unevaluable patients. We simulated latent failure and censoring times as follows:

For the latent failure times T, we considered three different hazard functions:

- A constant hazard function (an exponential distribution).
- An ascending monotone hazard function (a Weibull distribution with scale parameter equal to 2).
- A non monotone hazard function (a log-logistic distribution with a maximum of the hazard function reached on $\frac{t_{0}}{2}$).
  The shape parameter was determined by the magnitude of the response rate.

Latent censoring times C, were independently generated from either:

- A uniform distribution (*C* ∼ *U*_[0, *λ*]_).
- An exponential distribution (*C* ∼ *ɛ*(*λ*)).

Values for *λ* were obtained in order to have a pre-defined percentage of censoring *θ* at *t*_0_.

#### Configuration simulation

Sample sizes were obtained using an optimal Simon’s plan with (*π*_0_, *π*_1_, *α*, *β*) parameters. For each patient in each simulation, one failure time *T* and one censoring time *C* were simulated according to:

- The proportion of unevaluable patients at *t*_0_ in the trial was fixed at level *θ*;
- The response rate estimated at *t*_0_ in the trial was fixed to *π* such as: [1 − *F*(*t*_0_)] = *π*.

Several response rates at *t*_0_ have been simulated varying from 0.1 to 0.9 by 0.025.

Each configuration can be summarized by the parameters: *π*_0_, *π*_1_, *α*, *β*, *θ* and ${\tilde{t}}_{0}$. In order to mimic some intermediate evaluation time-points, we consider different values for ${\tilde{t}}_{0}$. One hundred thirty five configurations were simulated with each of the six combinations of distributions (exponential, Weibull, log-logistic failure time distribution and uniform and exponential distribution for censoring times) and combining the following parameters:

- *π*_0_ varying from 0.1 to 0.5 by 0.1;
- *π*_1_ = *π*_0_ + 0.2;
- (*α*, *β*) = {(0.05, 0.05); (0.1, 0.05); (0.1, 0.1)};
- *θ* = {0, 0.05, 0.20};
- ${\tilde{t}}_{0}=\left\{\frac{t_{0}}{4},\frac{t_{0}}{2},\frac{t_{0}}{4}\right\}$.

For each response rate in each configuration, 2000 single-arm phase II trials were simulated. Each trial was analyzed with the three *ad hoc* strategies and the *rescue* strategy (with either exponential or Weibull distributional assumption as stated in the previous section).

To evaluate the performance of the different strategies, we estimated the probability of stopping a trial for efficacy (*A*(*π*)), the type I error rate (*A*(*π*_0_)), the power (*A*(*π*_1_)) and the bias of the estimator of the true response rate (*π*). This latter quantity was estimated by: $\frac{1}{M}\overset{M}{\underset{m=1}{⅀}}({\hat{\pi }}_{m}-\pi )$ with *M* = 2000 and where ${\hat{\pi }}_{m}$ is the observed proportion of responders.

For the sake of clarity, we discussed thoroughly the results obtained with *π*_0_ = 0.3, *π*_1_ = 0.5, *α* = *β* = 0.1, *t*_0_ = 2, ${\tilde{t}}_{0}=\frac{t_{0}}{2}$ and the Weibull *rescue* strategy. The results obtained for the other configurations are just summarized in Table 1.

**Table 1:** Simulation results of the Weibull *rescue* strategy using Weibull failure times and uniform censoring times.

${\tilde{t}}_{0}=\frac{t_{0}}{2}$ and *θ* = 20%						
*π*_0_	*π*_1_	*α*	1 − *β*	*t*_0_	*A*(*π*_0_)	*A*(*π*_1_)
**0.3**	**0.5**	**0.1**	**0.9**	**2**	**0.108**	**0.878**
0.1	0.3	0.1	0.9	2	0.119	0.863
0.5	0.7	0.1	0.9	2	0.109	0.868
0.3	0.5	0.05	0.95	2	0.074	0.946
0.3	0.5	0.1	0.95	2	0.114	0.938
0.3	0.5	0.1	0.9	1	0.114	0.890

### Simulation results

**Fig 1:**
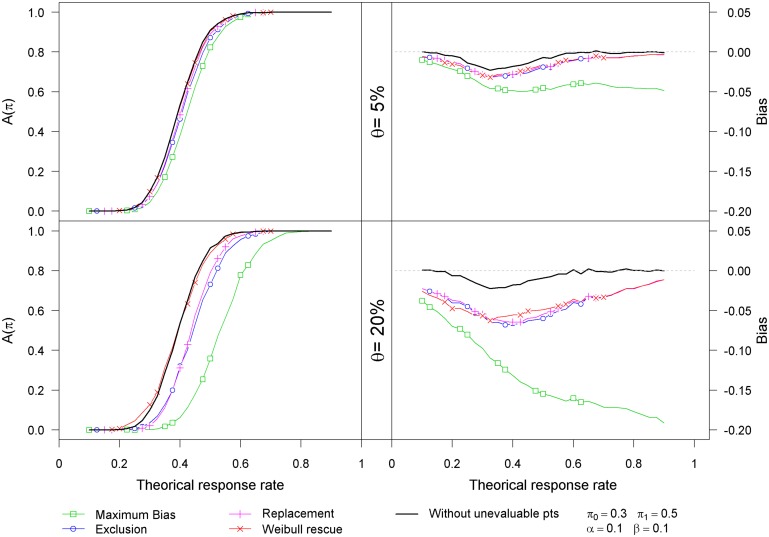
Results of the simulation study using Weibull failure times and uniform censoring times on Simon’s optimal design.

Fig 1 displays the impact of the proportion of unevaluable patients (5% and 20%) both for the bias (right panels) and the probability of stopping the trial for efficacy (left panels), with Weibull failure time distribution and uniform censoring time distribution.

As seen from the right panels, each strategy leads to a biased estimate of the response rate that increases with the proportion of unevaluable patients. The investigated strategies lead to negatively biased estimates. As the proportion of unevaluable patients increases, the *maximum bias* strategy shows dramatic increase in the bias of the estimator of the response rate. As seen in Table 2 (for 20% of unevaluable patients and a response rate of 0.4), the bias for the *rescue* strategy is close to those observed for the *replacement* and the *exclusion* strategy. As an example, for a Weibull failure time distribution and an uniform censoring distribution, the observed bias is −0.054 for the *replacement* and −0.056 for the *exclusion* strategies and −0.044 for the *rescue* strategy.

**Table 2:** Bias obtained by each strategies according to the simulated data distributions on optimal Simon’s design with *π* = 40%.

*π*_0_ = 0.3, *π*_1_ = 0.5, *α* = 0.1, *β* = 0.1, *θ* = 0.2, *t*_0_ = 2 and ${\tilde{t}}_{0}=\frac{t_{0}}{2}$						
	Maximum bias	Exclusion	Replacement	Weibull rescue	Exponential rescue	Without unevaluable patients
EU	−0.141	−0.079	−0.077	−0.068	−0.071	−0.017
EE	−0.135	−0.070	−0.068	−0.059	−0.060	−0.017
WU	−0.124	−0.056	−0.054	−0.044	−0.047	−0.018
WE	−0.122	−0.052	−0.049	−0.042	−0.042	−0.019
LU	−0.133	−0.069	−0.067	−0.059	−0.062	−0.016
LE	−0.132	−0.066	−0.065	−0.056	−0.059	−0.018

EU: exponential failure time and uniform censoring times, EE: exponential failure times and exponential censoring times, WU: Weibull failure times and uniform censoring times, WE: Weibull failure times and exponential censoring times, LU: log-logistic failure times and uniform censoring times, LE: log-logistic failure times and exponential censoring times.

The plots of the probability of stopping the trial for efficacy as a function of the response rate show the good behavior of the *rescue* strategy (left panels of Fig 1). For 5% of unevaluable patients, with the exception of the *maximum bias* strategy, the other strategies show similar behavior. When the proportion of unevaluable patients is 20%, only the *rescue* strategy performs adequately.

The estimated type I and type II error rates for the different configurations show that none of the strategies were able to meet the original requirements. As seen in Table 3 (with 20% of unevaluable patients), for the *replacement* strategy, the *exclusion* strategy and the *maximum bias* strategy, the results show conservative rates for the type I error associated with major power losses. The *maximum bias* strategy shows dramatic reduction of power with observed values that do not exceed 42%. For the other strategies, 20% of unevaluable patients and different simulations schemes, power results are around 75% for the *exclusion* strategy, 80% for the *replacement* and 85% for the *rescue* strategy. Thus, as compared to the *ad hoc* strategies, the *rescue* strategy offers a reasonable solution for maintaining type I and type II error rates as close as possible to the original requirements.

**Table 3:** Type I error rate and power provided by each strategy according to the simulated data distributions on optimal Simon’s design.

*π*_0_ = 0.3, *π*_1_ = 0.5, *α* = 0.1, 1 − *β* = 0.9, *θ* = 0.2, *t*_0_ = 2 and ${\tilde{t}}_{0}=\frac{t_{0}}{2}$							
		Maximum bias	Exclusion	Replacement	Weibull rescue	Exponential rescue	Without unevaluable patients
EU	*A*(*π*_0_)	0.001	0.023	0.015	0.135	0.103	0.099
	*A*(*π*_1_)	0.308	0.717	0.756	0.881	0.873	0.896
EE	*A*(*π*_0_)	0.003	0.028	0.018	0.129	0.109	0.093
	*A*(*π*_1_)	0.347	0.723	0.773	0.888	0.877	0.889
WU	*A*(*π*_0_)	0.003	0.044	0.034	0.108	0.166	0.101
	*A*(*π*_1_)	0.406	0.771	0.818	0.878	0.904	0.898
WE	*A*(*π*_0_)	0.003	0.059	0.044	0.126	0.183	0.102
	*A*(*π*_1_)	0.423	0.782	0.847	0.885	0.916	0.905
LU	*A*(*π*_0_)	0.004	0.036	0.026	0.151	0.152	0.107
	*A*(*π*_1_)	0.342	0.728	0.772	0.838	0.823	0.909
LE	*A*(*π*_0_)	0.001	0.036	0.021	0.156	0.160	0.100
	*A*(*π*_1_)	0.359	0.731	0.794	0.843	0.833	0.917

EU: exponential failure time and uniform censoring times, EE: exponential failure times and exponential censoring times, WU: Weibull failure times and uniform censoring times, WE: Weibull failure times and exponential censoring times, LU: log-logistic failure times and uniform censoring times, LE: log-logistic failure times and exponential censoring times.

In this work, two different distributions of latent censoring times and three different distributions of latent failure times were simulated. Whatever the distribution of latent failure times and censoring times, the investigated strategies have a similar behavior but the order of magnitude of the performance differs. As seen from the results (see details and Figure A in S1 File), the response rate estimation is quite sensible to choice of the distribution. As an example, for 20% of unevaluable patients, a true response rate of 40% and an uniform censoring distribution, the observed bias obtained with the *rescue* strategy is −0.044 when the latent failure times are Weibull distributed and −0.059 when failure times are log-logistic (see Table 2).

**Fig 2:**
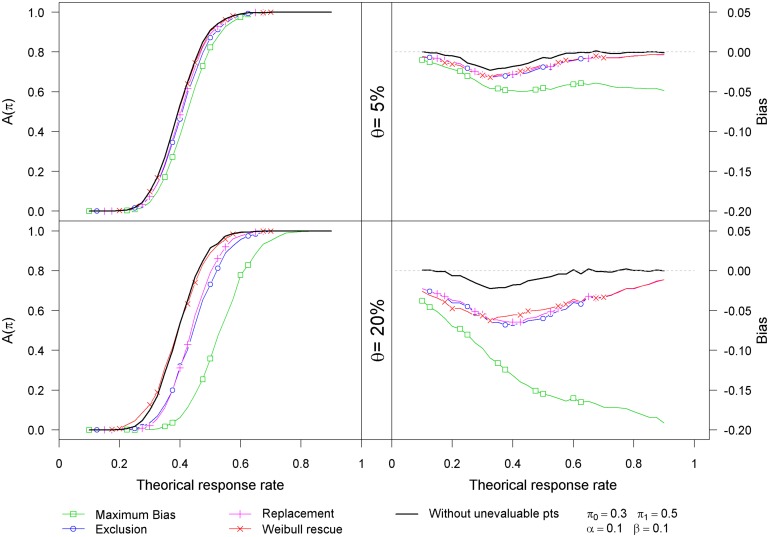
Results of the simulation study using Log-logistic failure times and exponential censoring times on Simon’s optimal design.

Fig 2 displays the impact of the proportion of unevaluable patients on the bias and the probability of stopping the trial for efficacy for log-logistic failure time and exponential censoring time distributions. The results are close to those displayed on Fig 1. As the *rescue* strategy relies on some distributional assumptions, their results are quite sensible to departure from these assumptions. Nevertheless, whatever the chosen hazard function, when the proportion of unevaluable patients is 20%, the *rescue* strategy leads to small deviations in terms of type I and type II error rates (see Table 3).

In order to evaluate the sensitivity of the Weibull *rescue* strategy when using an actuarial estimation obtained at two intermediate evaluations $\frac{t_{0}}{2}$ and *t*_0_ (see Section Simulation protocol), we performed additional simulations. We apply the rescue method with either $\frac{t_{0}}{4}$ or $\frac{3}{4}t_{0}$ as the first time-point. As seen from Table 4, the performance of the rescue strategy remained quite stable. As an example, assuming 20% of unevaluable patients, the type I error rate was 0.104 and the power was 87% when the first response evaluation is performed at $\frac{t_{0}}{4}$.

**Table 4:** Simulation results of type I and type II error rates obtained with the Weibull *rescue* strategy using Weibull failure times and uniform censoring times according to the visits calendar.

*π*_0_ = 0.3, *π*_1_ = 0.5, *α* = 0.1, 1 − *β* = 0.9 and *t*_0_ = 2			
		*θ* = 5%	*θ* = 20%
${\tilde{t}}_{0}=\frac{t_{0}}{4}$	*A*(*π*_0_)	0.089	0.104
	*A*(*π*_1_)	0.889	0.873
${\tilde{t}}_{0}=\frac{t_{0}}{2}$	*A*(*π*_0_)	0.088	0.108
	*A*(*π*_1_)	0.887	0.878
${\tilde{t}}_{0}=\frac{3}{4}t_{0}$	*A*(*π*_0_)	0.095	0.107
	*A*(*π*_1_)	0.890	0.879

When only one evaluation time-point is available, the Weibull *rescue* strategy could not be implemented and the computation of the new stopping boundaries are based on exponential assumption. The exponential *rescue* strategy performed well when the proportion of unevaluable patients is low (data not shown). As seen in Table 2, when the proportion of unevaluable patients is 20%, bias of the exponential *rescue* strategy is close to the one estimated with the Weibull *rescue* strategy. Nevertheless, type I error rate and type II error rate are inflated (see Table 3).

As discussed in section: The-proposed-strategy, various error rate functions could be implemented. An alternative error rate function ϕ* was also implemented in order to preserve type I error rate and concede an increase of type II error rate. Table 5 presents type I and type II error rates conceded according to the error rate function. The error rate function ϕ* preserves the type I error rate but the inflation of type II error rate is higher than the one conceded with the error rate function *ϕ*.

**Table 5:** Simulation results obtained with the Weibull *rescue* strategy using Weibull failure times and uniform censoring times according to the error rate function.

*π*_0_ = 0.3, *π*_1_ = 0.5, *α* = 0.1, 1 − *β* = 0.9 and *t*_0_ = 2			
*θ*	Error rate function	*A*(*π*_0_)	*A*(*π*_1_)
5%	*ϕ*	0.088	0.887
5%	ϕ*	0.087	0.898
20%	*ϕ*	0.108	0.878
20%	ϕ*	0.093	0.867

Finally, the impacts related to different values of *π*_0_, *π*_1_, *α*, *β* and *t*_0_ on the performance of each strategy are negligible (see Table 1). The configuration in bold is the one which is presented in detail previously. These parameters change the sample size of the trial but they have no impact on the performance of each strategy.

## Application on real clinical dataset

In this section, we applied the four different strategies to re-analyze one single-arm phase II trials conducted at the Institut Curie.

### Description

This trial was designed to evaluate radiotherapy with or without a novel chemotherapy in breast cancer patients with brain metastasis. The primary endpoint was the objective response rate at six weeks. Response was evaluated by a single cerebral MRI at 6-weeks. Death or progression were considered as treatment failures. Here, we focused on the group of patients who received the experimental treatment (radiotherapy and chemotherapy). Although, originally planned with Fleming two-stage design, we redesigned it with a Simon’s plan.

For a one-arm trial planned with an optimal Simon’s design [2] with *π*_0_ = 0.3 and *π*_1_ = 0.5, *α* = 0.1, *β* = 0.09 and a maximum sample size of 42 patients, we would have to consider the following rule: after the first stage, twenty patients would have been enrolled, if five or less objective responses were observed, the treatment would have been considered as ineffective and the trial would have been stopped. If more than five objective response were observed, additional patients are included. At the end of the second stage, forty-two patients would have been enrolled, if sixteen or less objective responses were observed, the treatment would have been considered ineffective. If more than sixteen objective responses were observed, the treatment would have been considered as showing potential activity of the combination and worthy of further study.

### Results

During the first stage, we observed eight objective responses, six non-responses, three deaths and three unevaluable patients. During the second stage, we observed thirteen objective responses, thirteen non-responses, one progression, nine deaths and six unevaluable patients. With our proposed *rescue* strategy relying on an exponential distribution, the stopping boundaries were 4 and 12 taking into account the number of unevaluable patients at the first and the second stage.

As presented in Table 6, 15% of patients were unevaluable during the first stage, each strategy led to an identical decision to proceed to the second stage. During the second stage, 14% of patients were unevaluable. The decisions differed between the strategies implemented. The three *ad hoc* strategies concluded that the treatment was ineffective. The *rescue* strategy concluded that the treatment was effective (see Table 6). Estimates of the six weeks response rate varied from 31% to 36%.

**Table 6:** Decisions that could be taken on real clinical trial data regarding the four strategies.

	First stage			Second stage		
	Observed objective response	Stopping boundary	Decision	Observed objective response	Stopping boundary	Decision
**Maximum Bias**	8	**5**	**Proceed to the second stage**	13	**16**	**Inefficacy**
**Exclusion**	8	**4**	**Proceed to the second stage**	13	**14**	**Inefficacy**
**Replacement**	8	**5**	**Proceed to the second stage**	15	**16**	**Inefficacy**
**Rescue strategy**	8	**4**	**Proceed to the second stage**	13	**12**	**Efficacy**

## Discussion

In this work, we investigated the performance of three different *ad hoc* strategies for handling unevaluable patients and proposed a novel so-called *rescue* strategy. This latter relies on computing new stopping boundaries that take into account the number of unevaluable patients observed at each stage. These new stopping boundaries used the conditional probabilities of responding at a time-point for an evaluable patient under the null and the alternative hypothesis.

As seen from the simulation, none of the *ad hoc* strategies can be recommended since they clearly lead to significant deviations from the planned constraints for type I and II error rates. In contrast, even though the *rescue* strategy does also not meet both type I and type II error rate requirements, it is the one which stays as close as possible to the planned error rates. As the *rescue* strategy makes some distributional assumptions, we evaluate its performance for departures from these assumptions. When the proportion of unevaluable patients is small, simulation results show that departures from distributional assumptions for the latent survival and censoring times do not markedly alter the performance of the strategy. When the proportion of unevaluable patients is high, type I error rate could be inflated which requires particular attention. In practice, when less than 10% of unevaluable patients are observed, whatever the distributional assumptions, the *res* cue strategy performed reasonably well (data not shown). With more than 10% of unevaluable patients, if distributional assumptions are questionable, the *rescue* strategy would not be recommend. However, if the distributional assumptions are tenable then the *rescue* strategy could be recommend.

A pragmatic way to conduct the analysis could be considered. Indeed, as the new stopping boundaries are always lower than the initial ones. If the number of observed responses among the evaluable patients $\overset{n_{j}-Z_{j}}{\underset{i=1}{⅀}}R_{i}$ is always greater than the initial stopping boundaries, no adaptation is needed to make the decision.

The motivation for the *rescue* strategy is that the sample size could not be modified during the trial according to the occurrence of unevaluable patients. Indeed, in practice, logistical aspects such as the number of subscription of patients insurance or ethical concerns prevent from modifying the sample size during the trial. Roughly speaking, the proposed *rescue* strategy allows to save what can be saved into a clinical trial with many unevaluable patients. In some cases, the proportion of unevaluable patients could be pre-specified and more subjects could be enrolled in order to obtain enough evaluable patients. This solution does not however prevent having more unevaluable patients than expected. Moreover, if the number of unevaluable patients has been overestimated it raises some ethical issues since more patients than necessary have been exposed to a potentially ineffective or harmful drug.

The use of the proposed *rescue* strategy in our clinical dataset highlights its practical interest in situations where 14% of the patients were unevaluable. The non-evaluability is related to the fact that the main criterion was shrinkage of the tumor burden evaluated by MRI. For some patients the disease has been rapidly progressive with the appearance of other metastatic sites requiring urgent treatment that led to postpone or cancel MRI. In such case, the occurrence of such clinical event which requires urgent care give less priority to the evaluation of the planned biological outcome. It leads to the occurrence of unevaluable patients who provide no information about the chosen biological outcome.

In this work, we assume the independence of failure and censoring times. However, if all the unevaluable patients experience an early disease progression, this assumption is obviously violated and performance of the *rescue* strategy is not guaranteed. For situation with potential informative censoring, we cannot recommend a particular strategy. This problem, which is beyond the scope of this article, needs further work.

In the literature, some authors have also tackled the problem of unevaluable patients. Koyama and Chen [9] recently proposed a proper inference from Simon’s two-stage design. Their method could provide a new critical value in order to make correct decision regarding the null hypothesis even when the sample size in the second stage has changed. However, they assumed that the actual first stage sample size is the same as that planned which represents limitations in current practice. Green and Dahlberg [10] investigated decision-making when the actual sample size differs from the planned sample size. They focused on the difficulty in a multicentric trial to close after accrual of a specified sample size. They proposed to compute a new stopping boundary at the first stage assuming that the second stage sample size could be reach. However, in our context, this last assumption no longer holds. Chen and Ng [11] proposed a flexible design by defining a collection of two-stage designs with different first or second stage sample size. They applied Simon’s optimal or minimax criteria to their designs in order to minimize the number of patients tested on an ineffective drug. An extension was also proposed by Masaki *et al*. [12]. These flexible designs improve the properties by controlling type I and type II error rates but are limited to a deviation of two patients from the planned sample size. However, they assumed that departures from the planned sample size is not related to ineligibility or non-evaluability of the patients. Focusing on trials in which recruitment of patients is slower than expected, Wu and Shih [13] proposed a rescue design which allows concluding at the first stage even if the first stage sample size is not attained. They modified the initial design by including fewer patients than planned and provide a new stopping boundary at the second stage. However, if the modified sample size is not attained, no solution is proposed. This strategy may be effective when accrual is lower than expected but is less appropriate for addressing the issue of unevaluable patients.

In summary, the proposed *rescue* strategy represents a practical and original solution when a Simon phase II trial has not been performed as planned. The strategy is simple to implement without requiring much patients and can be recommended for handling the occurrence of unevaluable patients. We plan to adapt the proposed method to Fleming’s designs [3].

## Supporting Information

S1 Text Details on formula (1). (PDF)

S1 File Impact of censoring and failure times distribution on response rate estimation in Simon’s two-stage designs. Bias on response rate estimation according to the latent failure times and censoring distribution (Figure A).
(PDF)

S2 File Scripts of R functions. (PDF)
