# Supplementary material for: A Rescue Strategy for Handling Unevaluable Patients in Simon’s Two Stage Design
Source: PLoS One. 2015 Sep 14;10(9):e0137586. doi: 10.1371/journal.pone.0137586 (PMC4569274; doi:10.1371/journal.pone.0137586)
Supplement: S1 Text — (PDF) [file pone.0137586.s001.pdf]

S1 File. Impact of censoring and failure times distribution on response rate estimation in Simon's two-stage designs

Notations were described in the article.

To show the impact of censoring and latent failure times distribution on response rate estimate at t_0 when unevaluable patients appear, we introduced the following equation using, for example, the exclusion strategy.

$$\hat{\pi}_j = \frac{X_j}{n_j - Z_j} \simeq n_j \left(1 - \frac{P\left(\{T < C\} \cap \{T < t_0\}\right)}{1 - P\left(\{C < T\} \cap \{C < t_0\}\right)}\right)$$

where : $P\left(\{T < C\} \cap \{T < t_0\}\right)$the probability of not responding at t_0 and

$P\left(\{C < T\} \cap \{C < t_0\}\right)$, the probability of being unevaluable at t_0.

We have used F and G the cumulative distribution functions of T and C respectively and f and g the respectives density functions.

$$P\left(\{T < C\} \cap \{T < t_0\}\right) = \int_0^{t_0} F(c)g(c)dc + F\left(t_0\right)\left(1 - G\left(t_0\right)\right)$$

and

$$P\left(\{C < T\} \cap \{C < t_0\}\right) = \int_0^{t_0} G(c)f(c)dc + G\left(t_0\right)\left(1 - F\left(t_0\right)\right)$$

Finally, equation is equivalent to ,

$$\begin{aligned} \hat{\pi}_j &\simeq n_j \left\{1 - \frac{\int_0^{t_0} F(c)g(c)dc + F\left(t_0\right)\left[1 - G\left(t_0\right)\right]}{1 - \int_0^{t_0} G(c)f(c)dc - G\left(t_0\right)\left[1 - F\left(t_0\right)\right]}\right\} \\ &\simeq n_j \left\{\frac{\left[1 - F\left(t_0\right)\right]\left[1 - G\left(t_0\right)\right]}{1 - \int_0^{t_0} G(c)f(c)dc - G\left(t_0\right)\left[1 - F\left(t_0\right)\right]}\right\} \end{aligned}$$

In this last equation, distributions have an impact on the response rate estimation at t_0.

Simulations have been performed to evaluate the impact of censoring distribution and the latent failure times distribution.

Fig A: Bias on response rate estimation according to the latent failure times and censoring distribution

EU: exponential failure time and uniform censoring times, EE: exponential failure times and exponential censoring times, WU : Weibull failure times and uniform censoring times, WE: Weibull failure times and exponential censoring times, LU : log-logistic failure times and uniform censoring times, LE: log-logistic failure times and exponential censoring times.

Fig A displayed that an uniform censoring lead to larger bias than exponential censoring whatever the latent failure times distribution. Moreover, exponential latent

failure times lead to a larger bias than loglogistic distribution or Weibull distribution. These results are true whatever the theorical response rate (data not shown) and whatever the unevaluable patients rate. Nevertheless, the more the unevaluable rate is the more the bias is important.
