# Supplementary material for: A Rescue Strategy for Handling Unevaluable Patients in Simon’s Two Stage Design
Source: PLoS One. 2015 Sep 14;10(9):e0137586. doi: 10.1371/journal.pone.0137586 (PMC4569274; doi:10.1371/journal.pone.0137586)
Supplement: S2 File — (PDF) [file pone.0137586.s003.pdf]

S2 File. Scripts of R functions.

1. R code to compute Optimal Simon design

```
#######
### Optimal.Simon.Plan allow to compute an Optimal two stage Simon plan
### ARGUMENTS :
### Here we denote
# pi0 :  the minimum expected efficacy of the treatment
# pi1 :  the desirable target level of treatment efficacy
# alpha:  the type I error rate
# beta:  the type II error rate
# Nmax :  the maximum number of patients to be included in the Phase II trial
### VALUES :
# a list :
# $risk :  final Simon Optimal design
# $j :  stage
# $n:  the number of patients included at stage j (N1, N2)
# $r:  the stopping boundaries at stage j (r1, r2)
# $alpha :  the spending function of type I error rate
# $beta :  the spending function of type II error rate
# $pet0 :  probability of early termination under the null hypothesis
# $pet1 :  probability of early termination under the alternative hypothesis
# $design:  all the selected design satisfing A(pi0)<alpha and A(pi1)>1-beta
Optimal.Simon.Plan<-function(pi0, pi1, alpha, beta, Nmax) {
r12<-expand.grid(list(r1=0:Nmax, r2=0:Nmax))
n12<-r12;names(n12)<-c("n1", "n2")
n12<-n12[n12$n1+n12$n2<=Nmax & n12$n1>0 & n12$n2>0,]
dim(n12)
r12<-r12[r12$r1<r12$r2,]
dim(r12)
id<-0
```

```
N<-Nmax
enh0C<-Nmax
n12<-n12[order(runif(dim(n12)[1])),]
n<-dim(n12)[1]
nid<-0
while (n>0) {
n1<-n12$n1[1]
n2<-n12$n2[1]
pi12<-expand.grid(list(k1=0:n1, k2=0:n2))
pi12$pk1.0<-dbinom(pi12$k1, n1, pi0)
pi12$pk2.0<-dbinom(pi12$k2, n2, pi0)
pi12$pk1.1<-dbinom(pi12$k1, n1, pi1)
pi12$pk2.1<-dbinom(pi12$k2, n2, pi1)
pi12$pk12.0<-pi12$pk1.0*pi12$pk2.0
pi12$pk12.1<-pi12$pk1.1*pi12$pk2.1
r12c<-r12[r12$r1<n1 & r12$r2<n1+n2,]
r12c<-r12c[order(runif(dim(r12c)[1])),]
dim(r12c)[1]
r12c$beta1<-pbinom(r12c$r1, n1, pi1)
r12c<-r12c[r12c$beta1<beta,]
dim(r12c)[1]
r12c$pet1<-pbinom(r12c$r1, n1, pi0)
r12c$enh0C<-n1+(1-r12c$pet1)*n2
r12c<-r12c[r12c$enh0C<=enh0C,]
dim(r12c)[1]
nr12c<-dim(r12c)[1]
while (nr12c>0) {
r1<-r12c$r1[1]
r2<-r12c$r2[1]
beta1<-r12c$beta1[1]
enh0<-r12c$enh0C[1]
ind<-(pi12$k1>r1) & (pi12$k2>r2-pi12$k1)
alpha2<-sum(pi12$pk12.0[ind])
ind<-(pi12$k1>r1) & (pi12$k2<=r2-pi12$k1)
beta2<-sum(pi12$pk12.1[ind])
c(beta1, alpha2, beta2)
```

```
if (alpha2<=alpha & beta1+beta2<=beta) {
if (enh0<enh0C) {
id<-id+1
enh0C<-enh0
N<-n1+n2
d<-c(n1, n2, r1, r2, 0, beta1, alpha2, beta2, enh0, N)
if (id==1) {
design<-d
} else {
design<-rbind(design, d)
}
} else {
if (n1+n2<N) {
id<-id+1
N<-n1+n2
d<-c(n1, n2, r1, r2, 0, beta1, alpha2, beta2, enh0, N)
if (id==1) {
design<-d
} else {
design<-rbind(design, d)
}
}
}
r12c<-r12c[-1,];nr12c<-nr12c-1
} else {
if (alpha2>alpha) {
r12c<-r12c[r12c$r1!=r1 | (r12c$r1==r1 & r12c$r2>r2),]
}
if (beta1+beta2>beta) {
r12c<-r12c[r12c$r1!=r1 | (r12c$r1==r1 & r12c$r2<r2),]
}
nr12c<-dim(r12c)[1]
}
}
if (id>nid) {
n12<-n12[n12$n1<=floor(enh0C),]
```

```
n<-dim(n12)[1]
nid<-id
} else {
n12<-n12[-1,];n<-n-1
}
}
risk<-NULL
if (id>0) {
d<-design[id,]
design<-data.frame(design)
names(design)<-c("n1", "n2","r1", "r2", "alpha1", "beta1","alpha2",
"beta2", "EN", "n_cum")
# Loading the Calcul.Simon.Risk function is required
risk<-Calculation.Simon.Risk(d[1], d[3], d[2], d[4], pi0, pi1)
} else{
stop("No design found")
}
res<-list(risk=risk, design=design)
return(res)
}
#######
### Calculation.Simon. Risk allow to calculate Simon risk depending on
the quadruplet and pi0 and pi1
### ARGUMENTS :
# n1: the number of patients included at stage 1
# n2: the additional number of patients included at stage 2
# r1 : the stopping boundary at stage 1
# r2: the stopping boundary at stage 2
# pi0 : the minimum expected efficacy of the treatment
# pi1 : the desirable target level of treatment efficacy
### VALUES :
# a dataframe : : final Simon Optimal design
# $j : stage
# $n: the number of patients included at stage j (N1, N2)
# $r: the stopping boundaries at stage j (r1, r2)
# $alpha : the spending function of type I error rate
```

```
# $beta : the spending function of type II error rate
# $pet0 : probability of early termination under the null hypothesis
# $pet1 : probability of early termination under the alternative
hypothesis
Calculation.Simon.Risk<-function(n1, r1, n2, r2, pi0, pi1) {
k<-(r1+1):n1
pk1<-dbinom(k, n1, pi1)
pk0<-dbinom(k, n1, pi0)
beta1<-pbinom(r1, n1, pi1)
beta2<-sum(pk1*pbinom(r2-k, n2, pi1))
alpha2<-sum(pk0*(1-pbinom(r2-k, n2, pi0)))
pet01<-pbinom(r1, n1, pi0)
pet02a<-sum(pk0*pbinom(r2-k, n2, pi0))
pet02b<-sum(pk0*(1-pbinom(r2-k, n2, pi0)))
pet11<-pbinom(r1, n1, pi1)
pet12a<-sum(pk1*pbinom(r2-k, n2, pi1))
pet12b<-sum(pk1*(1-pbinom(r2-k, n2, pi1)))
res<-data.frame(j=1:2, n=c(n1, n2+n1), r=c(r1, r2), alpha=c(0, alpha2),
alphacum=c(0, alpha2), beta=c(beta1, beta2), betacum=cumsum(c(beta1,
beta2)))
res$pet0<-c(pet01, pet02a+pet02b)
res$pet1<-c(pet11, pet12a+pet12b)
return(res)
}
```

2. R code to simulate data

```
##########################
####### Simul.data : function which allow to simulate data
### ARGUMENTS :
# N: Total number of patients to simulate
# pi : the theorical response rate
# theta : the unevaluable patients rate
# t0 : the evaluation time point
# Tdistrib : the distribution of the latent failure times T;
# W correspond to a Weibull distribution
# E correspond to an Exponential distribution
```

```
# L correspond to a Log logistic distribution
# Cdistrib :  the distribution of the censoring times C;
# U correspond to an Uniform distribution
# E correspond to an Exponential distribution
### VALUES :
# a data frame
# $time:  the time between inclusion and response evaluation or last
known contact
# $X : a dummy variable which indicates if patients respond to the
therapy (X=1) ,
# do not respond (X=0) or is unevaluable (X=NA)
Simul.data<-function(N=100, pi=0.2, theta=0, t0=1, Tdistrib=c("W",
"L","E"), Cdistrib=c("U", "E")){
Tdistrib<-Tdistrib[1]
Cdistrib<-Cdistrib[1]
# Define the cumulative probability and the random generation for the C
distribution
if(Cdistrib=="U"){
G<-function(c, C){ifelse(c<C, c/C ,1)}
rg<-function(n, C){runif(n, 0, C)}
}
if(Cdistrib=="E"){
G<-function(c, C){1-exp(-C*c)}
rg<-function(n, C){rexp(n,C)}
}
# Define the cumulative probability and the random generation for the T
distribution, specify the distribution
if(Tdistrib=="L"){
scale<-2
shape<-t0*((pi/(1-pi))^(1/scale))
f<-function(t, shape, scale){
y<-(t/shape)^scale
y<-(y/((1+y)^2))*(scale/t)
return(y)
}
rf<-function(n, shape, scale){
U<-runif(n, 0,1)
```

```
return(((U/(1-U))^(1/scale))*shape)
}
}
if(Tdistrib=="W"){
scale<-2
shape<-t0*((-log(pi))^(-1/scale))
f<-function(t, shape, scale){
y<-dweibull(t, shape=scale, scale=shape)
return(y)
}
rf<-function(n, shape, scale){rweibull(n, shape=scale, scale=shape)}
}
if(Tdistrib=="E"){
shape<-(-log(pi))/t0
scale<-1
f<-function(t, shape, scale){
y<-dexp(t, rate=shape)
return(y)
}
rf<-function(n, shape, scale){rexp(n, rate=shape)}
}
#If there is no unevaluable patients :  do not introduce censoring
if(theta==0){
lambda<-Inf
}else{
#to determine the lambda parameter
fct<-function(C, t0, shape, scale, theta){ PdV(C, t0, shape, scale, f,
G)-theta}
lambda<-uniroot(fct,interval=c(0.01, 1000),t0=t0, shape=shape,
scale=scale, theta=theta)$root
}
#Random generation
T<-rf(N, shape, scale)
if(lambda==0){
C<-T*Inf
}else{
```

```
C<-rg(N, lambda)
}
#calculate the time
time<-pmin(T, C)
#classify each simulated patient as responder (X=1), non responder (X=0) or unevaluable patients (X=NA)
X<-ifelse(T>t0, 1,0)
X<-ifelse(C<T & C<t0, NA,X)
data<-data.frame(time=time, X=X)
return(data)
}
#############
#### analyze_data : count the number of unevaluable patients and the actuarial survival at lt0 and t0 at each stage
### ARGUMENTS :
# N1: number of patients included at the first stage
# N2 : number of patients included at the second stage
# data : a data frame with
# data$time : the time from inclusion to response evaluation
# data$X : a dummy variable with X=1: response, X=0 non response, X=NA : unevaluable
# t0 : the evaluation time point
# l : the time ratio (as described in the article)
### VALUES :
# a list
# $stage1 : result at stage 1
# $Z1 : number of unevaluable patient at stage 1
# $AC : actuarial survival estimated at stage 1
# $S1 : the number of response at stage 1 among the N1-Z1 evaluable patients
# $stage2 : result at stage 2
# $Z2 : number of unevaluable patient at stage 2
# $AC : actuarial survival estimated at stage 2
# $S2 : the number of response at stage 2 among the N2-Z2 evaluable patients
analyze_data<-function(N1, N2, data, t0, l=NULL){
#Select the appropriate number patient to analyze
```

```
N2<-min(N2, dim(data)[1])
data1<-data[1:N1,]
data2<-data[1:N2,]
#calculate the unevaluable patients
Z1<-as.numeric(table(data1$X, exclude=NULL)[3])
Z2<-as.numeric(table(data2$X, exclude=NULL)[3] )
S1<-as.numeric(table(data1$X, exclude=NULL)[2])
S2<-as.numeric(table(data2$X, exclude=NULL)[2] )
#Estimate the actuarial survival at l.t0 and t0
if(is.null(l)){
AC1<-NULL
AC2<-NULL
} else {
AC1<- AC.fct(data1$time, data1$X, t0, l)$Scum
AC2<- AC.fct(data2$time, data2$X, t0, l)$Scum
}
res1<-list(Z1=Z1, AC=AC1, S1=S1)
res2<-list(Z2=Z2, AC=AC2, S2=S2)
res<-list( stage1=res1, stage2=res2)
return(res)
}
#############
#### AC: function wich estimate the actuarial survivate rate at l.t0 and
t0
### ARGUMENTS :
# time:  time from inclusion to response evaluation
# response :  response=1:  responder, response=0 non responder and
response=NA : uneavluable patients
# t0 :  the evaluation time point
# l :  the time ratio (as described in the article)
### VALUES :
# a dataframe
# $ti :  beginning of the time interval
# $t(i+1) :  end of the time interval
# $ci :  number of censored subjects
# $di:  number of event (1-response)
```

```
# $ni:  number of patients at risk during the interval (ti , t(i+1))
# $Scond :  conditional survival during the interval (ti , t(i+1))
# $Scum :  actuarial survival estimated at t(i+1)
AC.fct<-function(time, response, t0, l) {
#time<-DATA$time; response<-DATA$X ; l<-1/2
event<-ifelse(is.na(response) , 0, 1-response)
inter<-unique(c(0, l*t0, t0))
n<-length(time)
nbi<-length(l)+1
#define time interval
i<-cut(c(-1,-1,time), c(-1,inter, Inf), right=F)
x<-table(i, c(0,1,event))
x<-x[-1,]
tab<-data.frame(ti=inter)
tab$"t(i+1)"<-c(inter[-1], "Inf")
#number of censored
tab$ci<-x[,1]
#number of event
tab$di<-x[,2]
#Patients at risk
tab$ni<-c(n, n-cumsum(tab$ci+tab$di)[-dim(tab)[1]])
#Conditional survival
tab$Scond<-(tab$ni-tab$ci*0.5-tab$di)/(tab$ni-tab$ci*0.5)
tab$Scond<-ifelse(is.na(tab$Scond) , 1 ,tab$Scond)
#Cumulative survival
tab$Scum<-cumprod(tab$Scond)
tab<-tab[-dim(tab)[1],]
return(tab)
}
```

3. R code to use rescue strategy because Z_1 and Z_2 unevaluable patients appear

```
#######
#### PdV function calculate the probability to be unevaluable at t0 when
F and G are specified
### ARGUMENTS :
```

```
# Here, C is the censoring distribution parameter
# t0 :  time point for therapeutic evaluation
# shape :  the shape parameter of the distribution of T
# scale :  the scale parameter of the distribution of T
# f is the probability density function of T
# G is the cumulative probability function of C
### VALUES : the probability of being unevaluable at t0
PdV<-function(C, t0, shape, scale, f, G){
f1<-function(t, C, shape, scale){f(t, shape, scale)*G(t, C)}
f2<-function(t, t0,C, shape, scale){f(t, shape, scale)*G(t0, C)}
I1<-integrate(f1,0,t0, shape=shape, scale=scale, C=C)$value
I2<-integrate(f2,t0,Inf, shape=shape, scale=scale, C=C, t0=t0)$value
I<-I1+I2
return(I)
}
##############################################
##### Function tau.fct :  calculate the probability of not responding to
the therapy and being evaluable at t0
### ARGUMENTS :
# C: is the censoring distribution(C) parameter
# t0 :  time point for therapeutic evaluation
#shape :  the shape parameter of the distribution of T
#scale :  the scale parameter of the distribution of T
#F et G the cumulative distribution function of T and C respectively
#g:  the probability density function of C
### VALUES :the probability of not responding to the therapy and being
evaluable at t0
tau.fct<-function( C, t0,shape, scale, F, G, g){
f1<-function(c,C,shape, scale){F(c, shape=shape, scale=scale)*g(c, C=C)}
I1<-(integrate(f1,0, t0, shape=shape, scale=scale, C=C)$value )
tau<-I1 + F(t0, shape=shape, scale=scale)*(1-G(t0, C=C))
return(tau)
}
#####Calculate type I error rate
### ARGUMENTS
# a:  vector of the stopping boundary at stage 1 and 2
```

```
# n:  vector of the number of patients included
# p :  the treatment efficacy
## VALUES : probability of rejecting the null hypothesis when treatment efficacy is p
binom_alpha=function(r, n=c(10, 10), p=0.2){
sum(dbinom((r[1]+1):n[1] , n[1], p) * (1-pbinom (r[2] - ((r[1]+1):n[1]), n[2] , p)))
}
#####Calculate type II error rate
### ARGUMENTS
# r:  vector of the stopping boundary at stage 1 and 2
# n:  vector of the number of patients included
# p :  the treatment efficacy
## VALUES : probability of not rejecting the null hypothesis when treatment efficacy is p
binom_beta=function(r, n=c(10, 10), p=0.2){
sum(dbinom((r[1]+1):n[1] , n[1], p) * (pbinom (r[2] - ((r[1]+1):n[1]), n[2] , p)))
}
#####
#### Adapt:  furnish an the adaptive design denote (N1, a1, N2, a2) AD as decsribed in the article
### ARGUMENTS :
#Z1 :  the number of unevaluable patients observed at stage 1
#Z2:  the number of unevaluable patients observed at stage 2, if stage 2 is not completed yet Z2 is equal to Z1.
#N1 :  the planned sample size at stage 1
#N2:  the planned sample size at stage 2
#pi0 :  the minimu expected efficacy of the treatment
#pi1 :  the desirable target level of treatment efficacy
#alpha:  the type I error rate
#beta:  the type II error rate
#bound:  for stage 2 adaptation, bound is s=the stopping boundary used at the first stage
#l :  the time ratio (as described in the article)
#AC : vector of actuarial estimation of response rate at l.t0 and t0
# (if length of AC is 1:  exponential distribution is assumed) otherwise weibull distribution is assumed)
```

```
#error_fct :  the error rate function
# phi1 :respect the ratio between alpha and beta, increase the type I and type II error rates
# phi2:  preserv the type I error rate and increase the type II error rates
### VALUES
#a list object
# $param :  design parameter used in order to establish the adapted design
# pi0a :  the pi0* probability of response when being evaluable at t0 and under H0
# pi1a :  the pi0* probability of response when being evaluable at t0 and under H1
# alpha :  the initial type I error rate
# beta :  the initial type II error rate
# Nmax:  the maximal number of patient to be included
# Z1 :  the number of unevaluable patients at the first stage
# Z2 :  the number of unevaluable patients at the second stage
# $desAD : the adapted design defined by the adapted quadruplet and final the type I and type II error rate
# N1AD: number of evaluable patient at stage 1
# r1AD: the stopping boundary at first stage with Z1 unevaluable patients
# N2AD: number of evaluable patient at stage 2
# r2AD: the stopping boundary at second stage with Z2 unevaluable patients
# alphaAD : final type I error rate to establish the adapted design
# betaAD : final type II error rate to establish the adapted design
Adapt<-function( Z1, Z2=Z1,N1, N2, pi0,pi1, alpha, beta,t0, bound=NULL ,l=l, AC=NULL, error_fct=c("phi1", "phi2")){
##gamma=scale
#h00=shape0
#h01=shape1
Z2<-max(Z1, Z2)
if(is.null(bound)){bound<-c(N1, N2)
}else{
if(length(bound)==1){
bound<-c(bound, N2)
}
```

```
}
# Adaptation at stage 1
if(Z2-Z1==0){
n1<-N1-Z1
n2<-N2-N1
theta<-Z1/N1
a<-expand.grid(list(r1=0:n1 , r2=0:n2))
}
# Adaptation at stage 2
if (Z2-Z1>0){
n1<-N1-Z1
n2<-N2-N1-(Z2-Z1)
theta<-Z2/N2
a<-expand.grid(list(r1=bound[1] , r2=0:n2))
}
G<-function(c, C){punif(c, 0, C)}
g<-function(c, C){dunif(c, 0,C)}
#Weibull adaptation
if(length(AC)==2){
f<-function(t,shape, scale){dweibull(t, shape=scale, scale=shape)}
F<-function(t,shape, scale){pweibull(t, shape=scale, scale=shape)}
#Find weibull parameter (h0 and gamma) (shape and scale respectively)
scale<- log(log(AC[2])/log(AC[1]))/(-log(l))
shape0<- t0/(-log(pi0))^(1/scale)
shape1<- t0/(-log(pi1))^(1/scale)
#Find uniform parameter (lambda)
fct<-function(C, t0, shape, scale, theta){ PdV(C, t0, shape, scale, f,
G)-theta}
lambda0<-uniroot(fct,interval=c(0.01, 1000),t0=t0, shape=shape0,
scale=scale, theta=theta)$root
lambda1<-uniroot(fct,interval=c(0.01, 1000),t0=t0, shape=shape1,
scale=scale, theta=theta)$root
#Calculate adapted pi1 and pi0 :  equation 5
pi1a<-1-(tau.fct( lambda1, t0,shape1, scale, F, G, g)/(1-theta))
pi0a<-1-(tau.fct( lambda0, t0,shape0, scale, F, G, g)/(1-theta))
}
#Exponential adaptation
```

```
if(is.null(AC)){
f<-function(t,shape, scale){dexp(t, rate=shape)}
F<-function(t,shape, scale){1-exp(-(t*shape))}
#Find weibull parameter (h0 and gamma) (shape and scale respectively)
scale<- 1
shape0<- (-log(pi0))/t0
shape1<- (-log(pi1))/t0
#Find uniform parameter (lambda)
fct<-function(C, t0, shape, scale, theta){ PdV(C, t0, shape, scale, f,
G)-theta}
lambda0<-uniroot(fct,interval=c(0.01, 1000),t0=t0, shape=shape0,
scale=scale, theta=theta)$root
lambda1<-uniroot(fct,interval=c(0.01, 1000),t0=t0, shape=shape1,
scale=scale, theta=theta)$root
#Calculate adapted pi1 and pi0 :  equation 5
pi1a<-1-(tau.fct( lambda1, t0,shape1, scale, F, G, g)/(1-theta))
pi0a<-1-(tau.fct( lambda0, t0,shape0, scale, F, G, g)/(1-theta))
}
#### Détermination of the new stopping boundaries
a$r2<-a$r1 + a$r2
a<-a[a$r2<=bound[2] & a$r1<=bound[1],]
a$alpha1<- 0
a$beta1<-pbinom(a$r1, n1, pi1a)
a$alpha2<-apply(a[, c(1,2)], 1, binom_alpha, n=c(n1, n2), p=pi0a)
a$beta2<-apply(a[, c(1,2)], 1, binom_beta, n=c(n1, n2), p=pi1a)
a$enho<-n1+(1-pbinom(a$r1 , n1, pi0a))*n2
a$alpha<-a$alpha1+ a$alpha2
a$beta<-a$beta1+a$beta2
#error rate function to control type I and type II error rates
if(error_fct=="phi1"){
coef<-beta/alpha
a$alphacout<-a$beta/coef
a$ma<-pmax(a$alpha, a$alphacout)
a$ma[a$ma<alpha]<-2
a<-a[order(a$ma, a$enho),] #minimize the increase error rates then
E(N|H0)
desAD<-data.frame(N1AD = N1-Z1 , r1AD=a$r1[1], N2AD=N2-Z2 ,
r2AD=a$r2[1], alphaAD=a$ma[1] , betaAD=a$ma[1]*coef)
```

```
}
if(error_fct=="phi2"){
a<-a[a$alpha<=alpha,]
a$beta[a$beta<beta]<-2
a<-a[order(a$beta, a$enho),] #minimize the increase error rates then E(N|H0)
desAD<-data.frame(N1AD = N1-Z1 , r1AD=a$r1[1], N2AD=N2-Z2 , r2AD=a$r2[1], alphaAD=a$alpha[1] , betaAD=a$beta[1])
}
param<-data.frame(pi0a=pi0a, pi1a=pi1a, alpha=alpha , beta=beta, Nmax=N2-Z2 , Z1=Z1, Z2=Z2)
res<-list(param=param, desAD=desAD)
return(res)
}
```
